# AI‐Assisted Recognition of Actual Ureteral and Pancreatic Injuries in Laparoscopic Colorectal Surgery: A Randomized Video Review Study

**DOI:** 10.1002/ags3.70267

**Published:** 2026-08-24

**Authors:** Shunjin Ryu, Teppei Kamada, Kai Neki, Asuka Kuwashima, Yuta Imaizumi, Shunsuke Nakashima, Yasuhiro Takano, Yasunobu Kobayashi, Yasuhiro Takeda, Ken Eto

**Affiliations:** ^1^ Department of Digestive Surgery Kawaguchi Municipal Medical Center Kawaguchi Saitama Japan; ^2^ Department of Surgery The Jikei University School of Medicine Tokyo Japan; ^3^ Department of Surgery Machida Municipal Hospital Tokyo Japan; ^4^ Anaut Inc Tokyo Japan

**Keywords:** artificial intelligence, colorectal surgery, EUREKA, intraoperative complications, ureteral injuries

## Abstract

**Aim:**

Artificial Intelligence (AI)‐assisted anatomical recognition may help surgeons recognize intraoperative adverse events missed during surgery. We evaluated whether AI‐enhanced videos improved recognition of ureteral and pancreatic injuries.

**Methods:**

In this two‐institution randomized video‐review study using historical laparoscopic colorectal surgery videos, 150 surgeons reviewed either EUREKA‐generated AI‐enhanced videos or unenhanced videos. Five videos were reviewed: three actual injury videos, including unrecognized injuries, and two clinically comparable no‐injury control videos. The primary outcome was correct injured‐organ recognition. Secondary outcomes included recognition within the predefined injury time‐code window and correct no‐adverse‐event judgment.

**Results:**

Seventy‐five surgeons were assigned to each group, with no significant baseline differences. AI‐enhanced videos improved ureteral injury recognition in Video A (40.0% vs. 22.7%; *p* = 0.022) and Video C (50.7% vs. 28.0%; *p* < 0.01). Time‐code window recognition was greater for Video A (20.0% vs. 5.3%; *p* < 0.01) and Video C (18.7% vs. 4.0%; *p* < 0.01). Pancreatic injury recognition in Video D did not differ overall (77.3% vs. 70.7%; *p* = 0.352) but was greater among surgeons without advanced laparoscopic certification (81.3% vs. 63.3%; *p* = 0.048). Correct no‐adverse‐event judgments did not differ between groups.

**Conclusion:**

AI‐generated anatomical highlighting improved surgeons' recognition of actual ureteral injuries under controlled video‐review conditions without increasing incorrect adverse‐event judgments. These findings provide proof‐of‐concept evidence that AI‐generated overlays may support timely recognition of clinically important intraoperative adverse events. Prospective intraoperative studies are needed to determine whether this recognition benefit facilitates earlier management, changes operative decision‐making, reduces complications, or improves patient outcomes.

**Trial Registration:**

UMIN‐CTR; UMIN ID: UMIN000059439

## Graphical abstract

AI‐generated anatomical highlighting improved surgeons' recognition of actual ureteral injuries during laparoscopic colorectal surgery, including recognition within predefined injury time‐code windows. No significant overall improvement was observed for pancreatic injury, and incorrect adverse‐event judgments did not increase in no‐injury videos. These findings provide proof‐of‐concept evidence that AI‐generated overlays may support timely recognition of clinically important intraoperative adverse events.

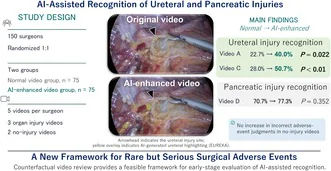

## 1 Introduction

Minimally invasive approaches are now widely used in gastrointestinal surgery, and intraoperative monitor images have become the dominant source of visual information for surgeons. In this environment, surgical safety depends not only on technical skill but also on accurate visual perception, attention, and interpretation of anatomy. Adverse surgical events are frequently associated with human performance deficiencies. Suliburk et al. reported that human performance deficiencies are identified in more than half of surgical adverse events and that a lack of recognition and a lack of attention are important execution‐related deficiencies [1]. In laparoscopic colorectal surgery, an anatomical structure may be visible on the monitor but still be insufficiently recognized by the surgeon. This gap between visibility and recognition may contribute to clinically important organ injury.

Ureteral injury during colorectal surgery is uncommon, with a reported incidence of 0.28%–1.5%; however, it is associated with prolonged hospitalization, increased morbidity and mortality, higher medical costs, long‐term renal impairment, and deterioration in quality of life related to ureteral stents or nephrostomy [2, 3, 4, 5, 6]. Delayed recognition is particularly problematic because postoperative diagnosis and delayed treatment are associated with hydronephrosis, ureteral stricture, renal dysfunction, and other long‐term sequelae [5, 6]. Preventive strategies, including fluorescent ureteral catheterization for high‐risk colorectal surgery, have been explored, but organ injury can still occur in both high‐risk and apparently routine cases [7]. Pancreatic injury during splenic flexure mobilization is also rare but can result in postoperative pancreatic fistula requiring fasting, antibiotics, percutaneous drainage, or reoperation [8]. Therefore, prevention must be accompanied by prompt intraoperative recognition and appropriate management.

Recent advances in surgical computer vision and artificial intelligence (AI) have created new opportunities to support intraoperative visual recognition [9, 10]. AI‐based systems have been developed to recognize surgical steps and key anatomical structures, including anatomical landmarks and safe or dangerous dissection zones, during minimally invasive surgery [11, 12].

EUREKA (Anaut Inc., Tokyo, Japan) is a deep‐learning‐based platform that analyzes surgical videos and generates color‐highlighted overlays of target anatomical structures. Previous EUREKA‐based studies have demonstrated the feasibility of highlighting the pancreas and neural structures during gastrointestinal surgery and have suggested potential educational value in laparoscopic colorectal surgery [13, 14, 15]. However, whether AI‐enhanced videos improve surgeons' recognition of actual intraoperative organ injury remains unclear. The present study aimed to evaluate whether AI‐enhanced EUREKA videos improved the recognition of actual ureteral and pancreatic injuries compared with standard unenhanced videos.

## 2 Methods

### 2.1 Study Design

**FIGURE 1:**
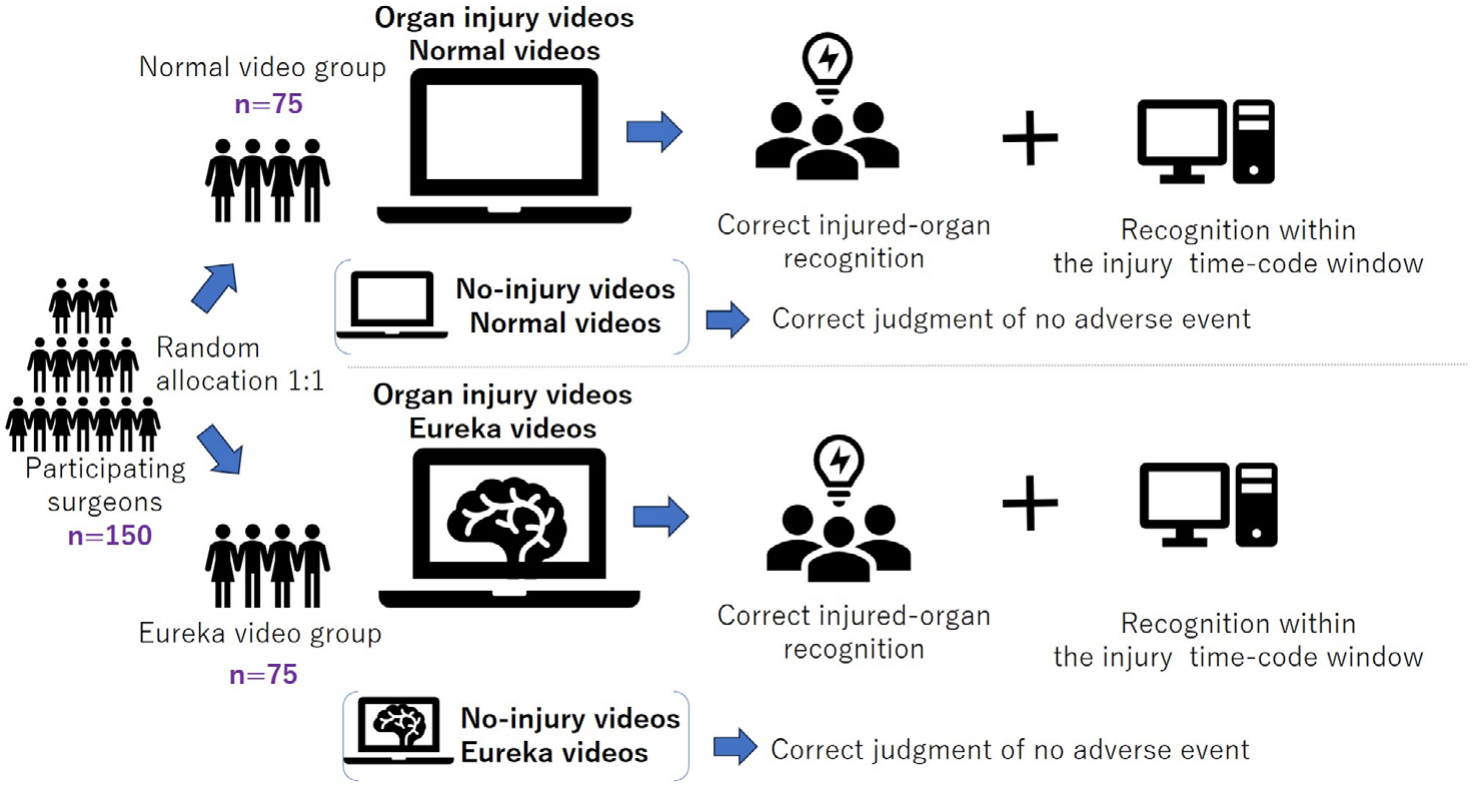
Study design of the randomized video review study. The participating surgeons were randomly assigned in a 1:1 ratio to either the Normal video group or the EUREKA video group. Each group reviewed organ injury videos and clinically comparable no‐injury control videos using either standard unenhanced videos or AI‐enhanced EUREKA videos. The outcomes were correct injured‐organ recognition, recognition within the predefined injury time‐code window, and correct judgment of no adverse events in no‐injury videos. All outcomes were compared between the Normal video group and the EUREKA video group.

This was a two‐institution, randomized video‐review study designed to evaluate whether AI‐enhanced surgical videos improve surgeons' recognition of intraoperative organ injury during laparoscopic colorectal surgery. Practicing surgeons were assigned to review either AI‐enhanced EUREKA videos or standard unenhanced videos (Normal videos) (Figure 1). The study used actual intraoperative injury scenes involving ureteral or pancreatic injury and clinically comparable no‐injury control scenes. Surgical videos were obtained from Kawaguchi Municipal Medical Center and Machida Municipal Hospital.

### 2.2 Participants and Group Allocation

Eligible participants were practicing gastrointestinal surgeons who were recruited through the investigators' professional and academic networks and who voluntarily participated in this study. Surgeons who had provided or edited the study videos were excluded from participation as reviewers. Participants were assigned to either the EUREKA video group or the Normal video group at a 1:1 ratio.

To balance baseline laparoscopic colorectal surgical experience between groups, participants were stratified according to whether they had performed fewer than 30 or 30 or more laparoscopic or robotic colorectal procedures as the primary surgeon before random allocation. This threshold was selected with reference to previous studies evaluating the learning curve for laparoscopic colorectal resection and laparoscopic low anterior resection [16, 17]. Random allocation was performed within each stratum using a random number sequence generated by the RAND function in Google Sheets (Google LLC, Mountain View, CA, USA).

The target sample size was pragmatically set at 150 surgeons, with 75 assigned to each group, on the basis of the expected number of eligible surgeons who could be recruited during the study period. No formal sample size calculation was performed.

### 2.3 Preparation of Study Videos

**FIGURE 2:**
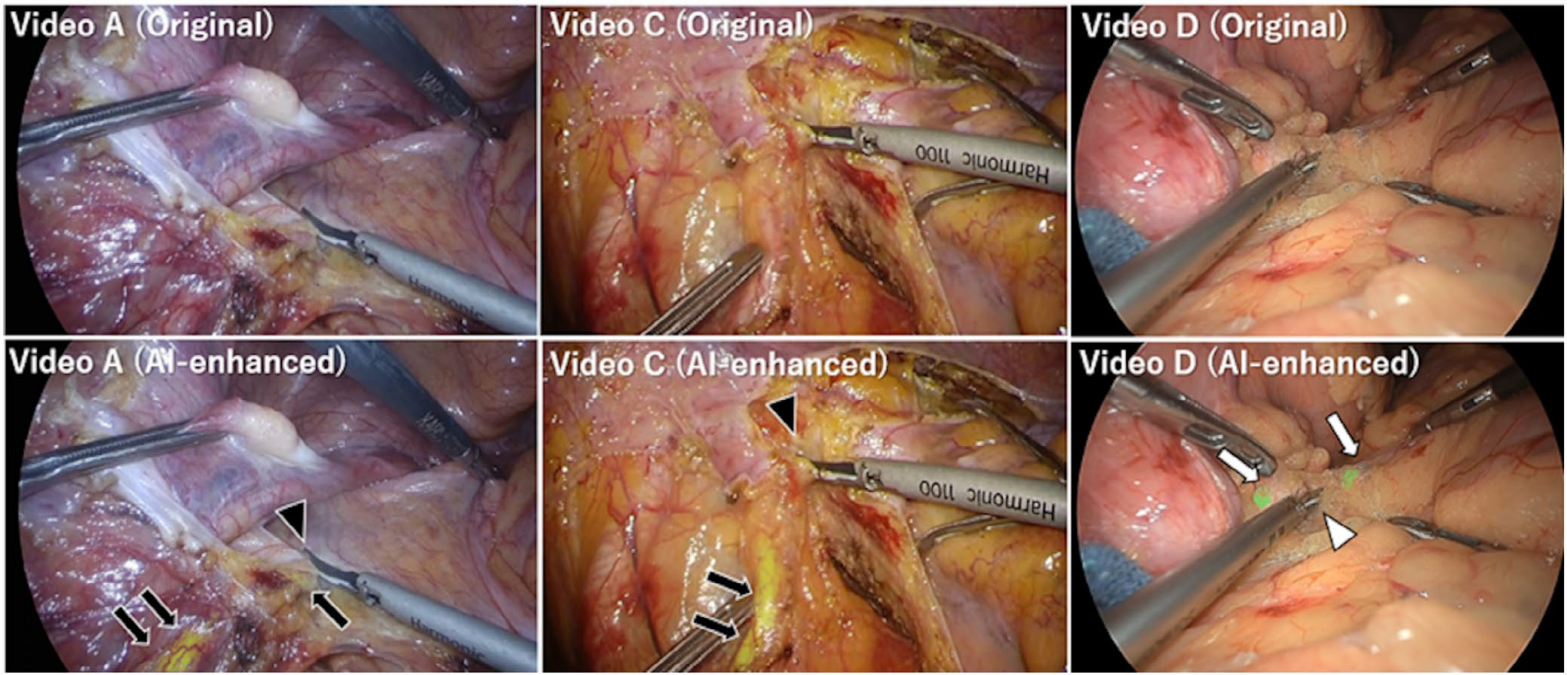
Representative organ injury scenes during the predefined injury time‐code windows. The columns show Videos A, C, and D, and the rows show the original and corresponding AI‐enhanced images. Black arrows indicate AI‐highlighted ureteral regions, and black arrowheads indicate ureteral injury sites. White arrows indicate AI‐highlighted pancreatic regions, and the white arrowhead indicates the pancreatic injury site.

Five laparoscopic colorectal surgery videos were prepared for review (Figure 2). Three videos included actual intraoperative organ injuries, and two videos were selected as no‐injury controls.

Video A showed a ureteral injury that occurred during rectal dissection in a sigmoid colon cancer case in which standard surgical dissection was considered feasible. The injury was not recognized intraoperatively and was diagnosed on postoperative day 2 as a Clavien–Dindo Grade IIIa complication requiring ureteral stent placement [18].

Video C showed a ureteral injury that occurred during difficult rectal dissection in a high‐risk rectal cancer patient with suspected invasion or dense adhesion to surrounding pelvic tissues on the basis of intraoperative findings. The injury was recognized in the later phase of the operation and was treated intraoperatively with uretero‐ureterostomy and ureteral stent placement.

Video D showed a pancreatic injury that occurred during splenic flexure mobilization in a patient with descending colon cancer in which standard surgical dissection was considered feasible. The injury was diagnosed postoperatively as a pancreatic fistula and was managed conservatively with fasting and antibiotics as a Clavien–Dindo Grade II complication.

Two additional videos were selected as clinically comparable no‐injury controls. Video B showed rectal dissection around the ureter without ureteral injury, and Video E showed splenic flexure mobilization around the pancreatic tail without pancreatic injury. The no‐injury videos were selected by consensus among three surgeons certified by the Endoscopic Surgical Skill Qualification System (ESSQS) as clinically comparable controls based on the operative field, surgical phase, and laparoscopic view. The ESSQS is a technical certification system established by the Japan Society for Endoscopic Surgery to evaluate laparoscopic surgical skills [19]. Representative original and AI‐enhanced images from Videos B and E are provided in Figure S1.

All study clips were anonymized, presented without audio, and edited to exclude repair procedures, ureteral stent placement, suturing of the injured site, and on‐screen text revealing the final diagnosis. For Video C, the subsequent scene in which the operating surgeon recognized the ureteral injury was also excluded from the study clip. Before the study, each of the three ESSQS‐certified surgeons individually reviewed each complete study video and considered the start and end points of the injury‐producing sequence. The three surgeons subsequently conducted a joint video review and determined the final predefined injury time‐code windows through discussion and consensus. Each window was defined not as a single instantaneous frame, but as the entire continuous sequence of causative maneuvers that directly resulted in organ injury. The final windows were determined by consensus during joint review; therefore, no formal inter‐rater agreement statistic was calculated. The window was 10 s for Video A, 18 s for Video C, and 10 s for Video D.

### 2.4 AI‐Enhanced Video Generation

AI‐enhanced videos were generated using EUREKA, a commercially available deep‐learning‐based surgical video analysis platform that creates color‐highlighted overlays of target anatomical structures. The ureter‐highlighted model was applied to Videos A–C, and the pancreas‐highlighted model was applied to Videos D and E. The same five operative scenes were used in both groups; only AI‐enhanced anatomical highlighting differed.

### 2.5 Video Review Procedure

Video review was conducted between July 1, 2025, and April 30, 2026, using a custom‐developed offline video review tool implemented in HTML and JavaScript. Before the review, all participants received standardized prerecorded instructions to minimize instructor‐related bias. Participants were informed that the five study videos included both videos without obvious intraoperative adverse events and videos containing adverse events that could lead to postoperative complications; they were not informed that every video contained an injury. Participants in the EUREKA group were additionally informed that the color overlay represented a region recognized by the AI as the ureter, pancreas, or nerve. Participants were instructed to pause the video at the moment they judged that an adverse event had occurred, rather than when they merely anticipated a possible event.

**FIGURE 3:**
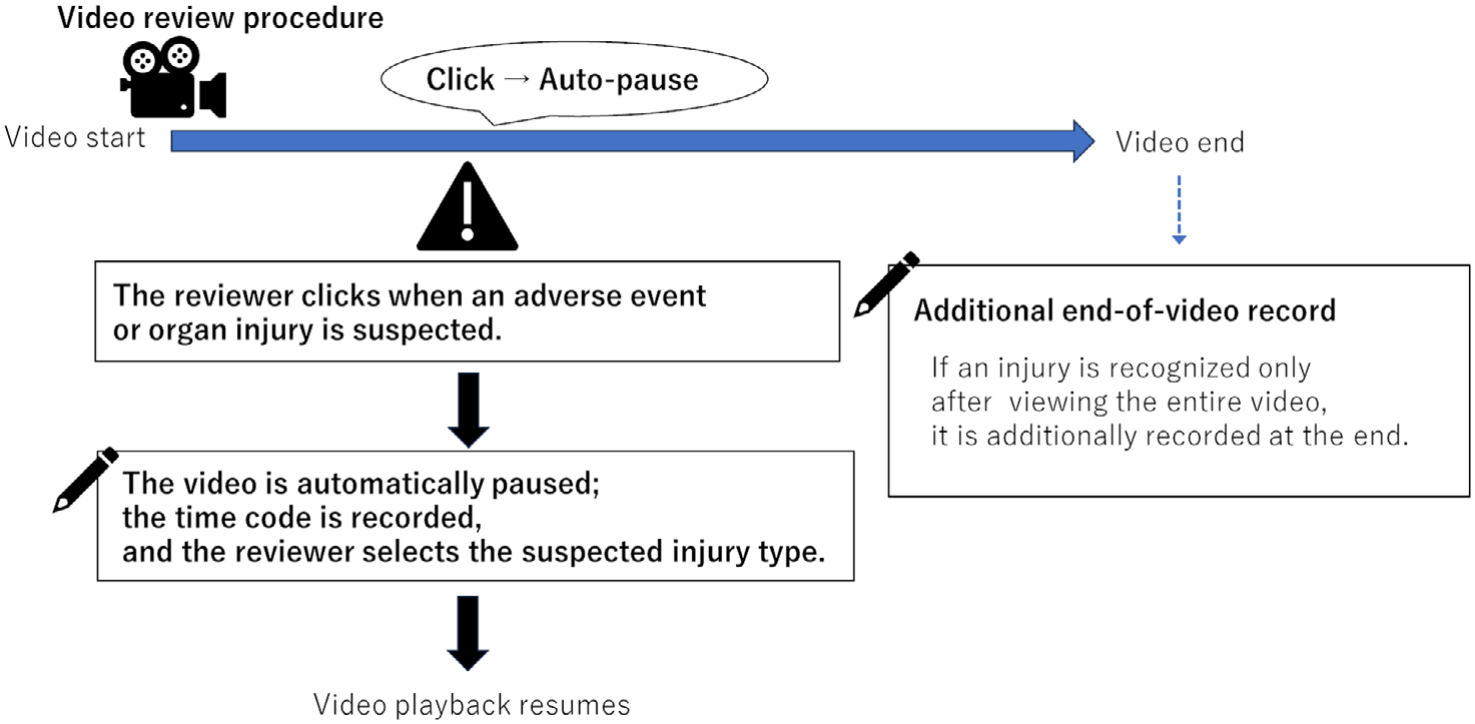
Video review and event‐recording procedure. During video review, reviewers clicked the video when they suspected an adverse event or organ injury. The video was automatically paused, the corresponding time code was recorded, and the reviewer selected the suspected injury type. Video playback then resumed. If an injury was recognized only after completion of the video, the suspected injury was additionally recorded at the end of the video.

Each participant reviewed five videos, each 2–3 min long, labeled with anonymized codes from A to E. The order of video presentation was randomized individually for each reviewer. Participants were allowed to watch each video only once at a standardized playback speed of 1.0×; rewinding and repeated viewing were not permitted. When participants judged that an adverse event or organ injury had occurred, they clicked on the video at that moment (Figure 3). The video was automatically paused, and the corresponding time code was recorded. Participants then selected the suspected type of injury from a predefined list of adverse events, including ureteral injury, pancreatic injury, vascular injury, nerve injury, bowel injury, and mesenteric injury. To approximate the open‐ended vigilance required in actual surgery and avoid constraining participants to the assumption that only one adverse event could occur, up to three distinct suspected adverse events could be recorded at different time points within each video. If participants could not identify the exact timing but recognized an injury after viewing the entire video, they could additionally record the suspected injury at the end of the video. All collected data, including timestamps and event categories, were exported as CSV files for subsequent statistical analysis.

Participant background information was collected, including years of clinical experience, surgical specialty, board certification status, ESSQS certification status, prior participation in a surgical video‐review study, and whether the participant had performed 30 or more laparoscopic or robotic colorectal procedures as the primary surgeon.

### 2.6 Outcomes

The primary outcome was the correct recognition rate of organ injury, which was assessed separately for each of the three predefined injury videos. Correct recognition was defined as the selection of the correct injured organ for each injury video, regardless of whether the response was recorded during video playback or after completion of video review.

Because the three injury videos represented clinically distinct scenarios—ureteral injury during an apparently routine colorectal cancer operation, ureteral injury during high‐risk rectal cancer surgery, and pancreatic injury during splenic flexure mobilization—each video was analyzed separately rather than pooled into a single composite endpoint.

Secondary outcomes included recognition within the predefined injury time‐code window for each injury video and correct judgment of no adverse events in the two no‐injury videos. Recognition within the predefined injury time‐code window required both correct identification of the injured organ and recording of the response within the predefined time‐code window during video playback. The percentages for this outcome were calculated using all the reviewers in each group as the denominator. Responses made only after completion of the video review were included in the primary analysis of organ injury recognition but were not counted as recognition within the predefined injury time‐code window.

For no‐injury videos, correct judgment of no adverse event was defined as completion of video review without selecting any adverse event or organ injury.

### 2.7 Statistical Analysis

Categorical variables are presented as numbers and percentages, and continuous variables are presented as medians with interquartile ranges. The baseline characteristics of the reviewers were compared between the EUREKA video group and the Normal video group using the chi‐squared test or Fisher's exact test for categorical variables, as appropriate, and the Mann–Whitney U test for continuous variables.

For each injury video, correct recognition and recognition within the predefined injury time‐code window were compared between groups. For the no‐injury videos, correct judgments of no adverse events were compared between groups. For the injury video outcomes, risk differences with 95% confidence intervals were calculated to estimate the magnitude of between‐group differences in recognition rates.

Exploratory subgroup analyses were performed according to ESSQS certification status. For each injury video, the interaction between the video group and ESSQS certification status was assessed using a logistic regression model with correct recognition of organ injury as the dependent variable and the video group, ESSQS certification status, and their interaction term as independent variables.

Post hoc sensitivity analyses were performed using only the first recorded response for each video and using injury time‐code windows expanded by 2 s at both the beginning and end. The primary and secondary outcomes, including separate evaluation of each injury video, were prespecified in the study protocol because the videos represented clinically distinct scenarios. No adjustment for multiplicity was performed. All statistical analyses were performed using STATA/IC version 16.0 (StataCorp, College Station, TX, USA), and two‐sided *p* values < 0.05 were considered to indicate statistical significance.

### 2.8 Ethical Considerations

This study was approved by the institutional review boards of Kawaguchi Municipal Medical Center (2024–46) and Machida Municipal Hospital (CT9‐2). This study was registered with the University Hospital Medical Information Network Clinical Trials Registry (UMIN‐CTR; UMIN ID: UMIN000059439). All surgical videos used in this study were obtained from cases for which consent for research and educational use had been obtained. All videos were anonymized before review to prevent patient identification.

Reviewer participation was voluntary and unpaid, and all the reviewers agreed to participate before the video review. Responses were analyzed anonymously.

## 3 Results

### 3.1 Reviewer Characteristics

A total of 150 surgeons participated in the video review and were randomly assigned to the EUREKA video group or the Normal video group, with 75 reviewers in each group. There were no missing data for reviewer characteristics or recognition outcomes across the five videos.

Baseline reviewer characteristics were well balanced between the two groups (Table 1). The median duration of clinical experience was 10 years in the EUREKA group and 9 years in the Normal video group. No significant differences were observed between groups in board certification status, ESSQS certification status, surgical specialty, or experience with 30 or more laparoscopic or robotic colorectal procedures as the primary surgeon.

**TABLE 1:** Comparison of the clinical experience and background characteristics of the surgeons performing the video review between the EUREKA video and Normal video groups.

Variables	Total or median	EUREKA group	Normal group	*p* value
Reviewers	150	75	75	
Clinical experience (years)	9.5 (5–15)	10 (5–15)	9 (5–15)	0.87
Board‐certified general surgeon by the JSS	126 (84.0%)	63 (84.0%)	63 (84.0%)	1.00
Board‐certified gastroenterological surgeon by the JSGS	94 (62.7%)	45 (60.0%)	49 (65.3%)	0.50
ESSQS‐certified surgeon by the JSES	53 (35.3%)	27 (36.0%)	26 (34.7%)	0.86
Specialty				0.35
Gastrointestinal surgery	105 (70.0%)	56 (74.7%)	49 (65.3%)	
Hepatobiliary and pancreatic surgery	14 (9.3%)	7 (9.3%)	7 (9.3%)	
Others	31 (20.7%)	12 (16.0%)	19 (25.3%)	
Laparoscopic colorectal surgery experience as primary surgeon, ≥ 30	97 (64.7%)	48 (64.0%)	49 (65.3%)	0.86
Prior participation in a surgical video‐review study	8 (5.3%)	3 (4.0%)	5 (6.7%)	0.47

*Note:* Values are presented as *n* (%) or median (interquartile range).

Abbreviations: ESSQS, Endoscopic Surgical Skill Qualification System; JSES, Japan Society for Endoscopic Surgery; JSGS, Japanese Society of Gastroenterological Surgery; JSS, Japan Surgical Society.

### 3.2 Video Review Outcomes

Across all reviewers, the numbers of reviewers who provided timestamped responses during video playback for the correct injured organ were 39 (26.0%) for Video A, 59 (39.3%) for Video C, and 100 (66.7%) for Video D.

The evaluation outcomes are shown in Table 2. With respect to Video A, which showed a ureteral injury during rectal dissection in a sigmoid colon cancer patient, the correct recognition rate was significantly greater in the EUREKA group than in the Normal video group (40.0% vs. 22.7%; risk difference, 17.3%; 95% CI, 2.9–31.8; *p* = 0.022). Recognition within the predefined injury time‐code window was also more frequent in the EUREKA group (20.0% vs. 5.3%; risk difference, 14.7%; 95% CI, 3.7–25.6; *p* < 0.01).

**TABLE 2:** Results of the video review analysis among all the reviewers.

Video	EUREKA group (*n* = 75)	Normal group (*n* = 75)	Risk difference, percentage points (95% CI)	*p* value
Video A (Ureteral injury during rectal dissection)				
Recognition	30 (40.0%)	17 (22.7%)	**+17.3 (2.9 to 31.8)**	**0.022**
Recognition within the predefined injury time‐code window	15 (20.0%)	4 (5.3%)	**+14.7 (3.7 to 25.6)**	**< 0.01**
Video B (No injury during rectal dissection)	66 (88.0%)	68 (90.7%)	—	0.59
Video C (Ureteral injury during difficult rectal dissection)				
Recognition	38 (50.7%)	21 (28.0%)	**+22.7 (7.4 to 37.9)**	**< 0.01**
Recognition within the predefined injury time‐code window	14 (18.7%)	3 (4.0%)	**+14.7 (3.3 to 26.0)**	**< 0.01**
Video D (Pancreatic injury during splenic flexure mobilization)				
Recognition	58 (77.3%)	53 (70.7%)	**+6.7 (−7.4 to 20.8)**	0.352
Recognition within the predefined injury time‐code window	24 (32.0%)	24 (32.0%)	**0.0 (−15.0 to 15.0)**	1.0
Video E (No injury during splenic flexure mobilization)	66 (88.0%)	60 (80.0%)	—	0.18

*Note:* Values are presented as the number of reviewers with the outcome and percentage. For the no‐injury videos, the values indicate correct judgment of no adverse events. Differences in risk with 95% CIs are shown for the injury video outcomes. Bold values indicate statistical significance (*p* < 0.05).

For Video C, which showed ureteral injury during difficult rectal dissection in high‐risk rectal cancer surgery, the correct recognition rate was significantly higher in the EUREKA group than in the Normal video group (50.7% vs. 28.0%; risk difference, 22.7%; 95% CI, 7.4–37.9; *p* < 0.01). Recognition within the predefined injury time‐code window was also significantly more frequent in the EUREKA group (18.7% vs. 4.0%; risk difference, 14.7%; 95% CI, 3.3–26.0; *p* < 0.01).

For Video D, which showed pancreatic injury during splenic flexure mobilization, the correct recognition rate did not differ significantly between the EUREKA and Normal video groups (77.3% vs. 70.7%; risk difference, 6.7%; 95% CI, −7.4 to 20.8; *p* = 0.352). Recognition within the predefined injury time‐code window was identical between groups (32.0% vs. 32.0%; risk difference, 0%; 95% CI, −15.0 to 15.0; *p* = 1.00).

In the two no‐injury videos, correct judgment of no adverse events did not differ significantly between groups.

**FIGURE 4:**
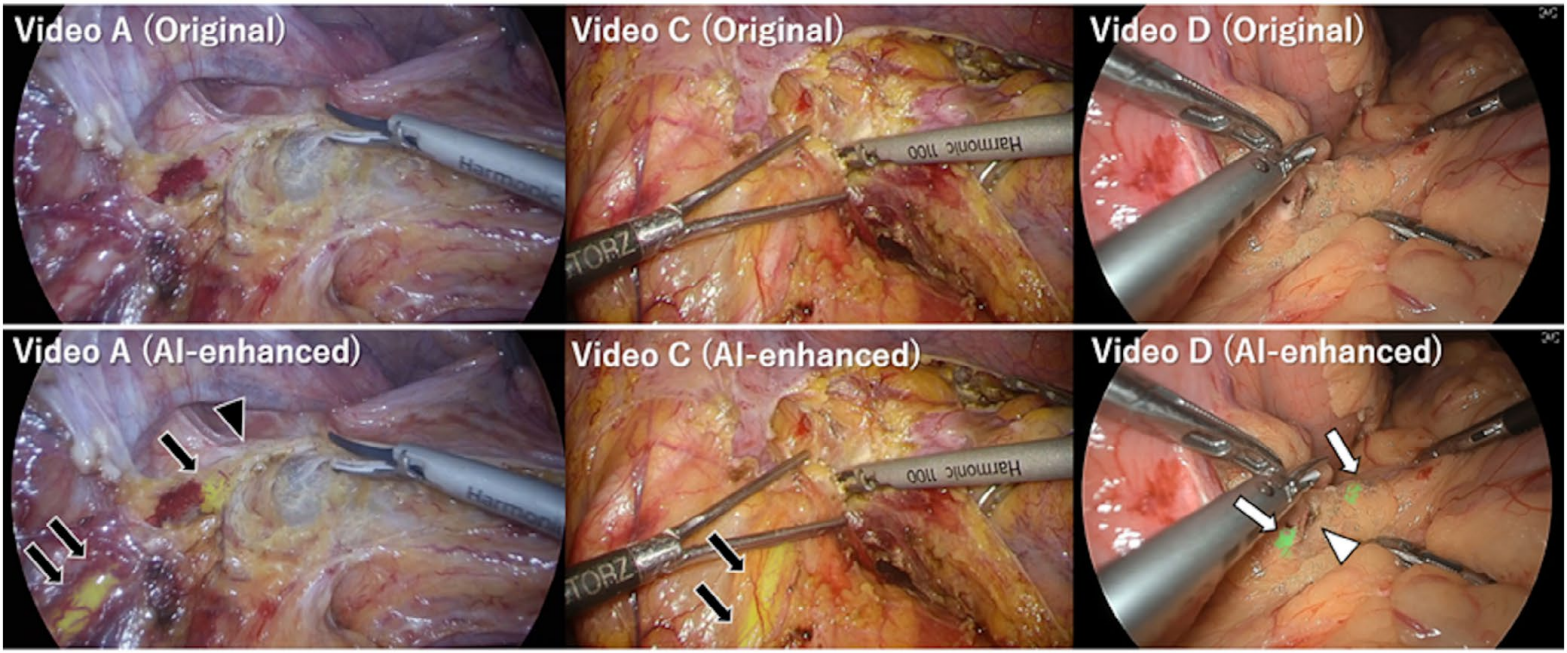
Representative visual cues potentially contributing to recognition after the predefined injury time‐code window. The columns show Videos A, C, and D, and the rows show the original and corresponding AI‐enhanced images. In Video A, urine leakage became visible from the thermally injured ureteral segment distal to the AI‐highlighted ureter. In Video C, no transected ureteral stump was visible; however, after release of tissue traction, an anatomical inconsistency between the highlighted ureteral course and the dissection plane became appreciable. In Video D, a cauterized area within the pancreatic parenchyma became visible after the injury‐producing maneuver. Black arrows indicate AI‐highlighted ureteral regions, and black arrowheads indicate ureter‐related post‐window visual cues. White arrows indicate AI‐highlighted pancreatic regions, and the white arrowhead indicates the pancreatic post‐window visual cue.

Representative visual findings available after the predefined injury time‐code windows are shown in Figure 4. In Video A, urine leakage became visible from the thermally injured ureteral segment distal to the AI‐highlighted ureter, although the injured segment itself was not directly highlighted. In Video C, no transected ureteral stump was visible; however, after release of tissue traction, an anatomical inconsistency between the highlighted ureteral course and the dissection plane became appreciable. In Video D, a cauterized area within the pancreatic parenchyma became visible after the injury‐producing maneuver.

In post hoc sensitivity analyses, the results for Videos A and C remained significant when recognition was restricted to the first recorded response and when the predefined injury time‐code windows were expanded by 2 s at both boundaries (Table S1). No significant between‐group difference was observed for Video D in either sensitivity analysis.

### 3.3 Exploratory Subgroup Analysis According to ESSQS Certification Status

In the exploratory subgroup analysis, no significant differences in recognition outcomes were observed between the EUREKA and Normal video groups among ESSQS‐certified surgeons for any of the five videos (Table 3).

**TABLE 3:** Subgroup analysis of the video review outcomes according to ESSQS certification status.

Video	EUREKA group	Normal group	*p* value
ESSQS‐certified surgeons	27	26	
Video A (Ureteral injury during rectal dissection)	6 (22.2%)	4 (15.4%)	0.52
Video B (No injury during rectal dissection)	24 (88.9%)	24 (92.3%)	0.67
Video C (Ureteral injury during difficult rectal dissection)	12 (44.4%)	9 (34.6%)	0.46
Video D (Pancreatic injury during splenic flexure mobilization)	19 (70.4%)	22 (84.6%)	0.21
Video E (No injury during splenic flexure mobilization)	24 (88.9%)	19 (73.1%)	0.14
Non‐ESSQS‐certified surgeons	48	49	
Video A (Ureteral injury during rectal dissection)	24 (50.0%)	13 (26.5%)	**0.017**
Video B (No injury during rectal dissection)	42 (87.5%)	44 (89.8%)	0.72
Video C (Ureteral injury during difficult rectal dissection)	26 (54.2%)	12 (24.5%)	**< 0.01**
Video D (Pancreatic injury during splenic flexure mobilization)	39 (81.3%)	31 (63.3%)	**0.048**
Video E (No injury during splenic flexure mobilization)	42 (87.5%)	41 (83.7%)	0.59

*Note:* Values are presented as the number of reviewers with the outcome and percentage. For the no‐injury videos, the values indicate correct judgment of no adverse events. Bold values indicate statistical significance (*p* < 0.05).

Abbreviation: ESSQS, Endoscopic Surgical Skill Qualification System.

Among non‐ESSQS‐certified surgeons, the EUREKA group had significantly higher correct recognition rates than the Normal video group did for Video A (50.0% vs. 26.5%; *p* = 0.017), Video C (54.2% vs. 24.5%; *p* < 0.01), and Video D (81.3% vs. 63.3%; *p* = 0.048). Correct judgment rates in the no‐injury videos did not differ significantly between groups among the ESSQS‐certified or non‐ESSQS‐certified surgeons.

Interaction analyses revealed no significant interaction between the video group and ESSQS certification status for Video A (interaction *p* = 0.498) or Video C (interaction *p* = 0.220). A significant interaction was observed only for Video D (interaction *p* = 0.035), suggesting a possible difference in the effect of EUREKA video guidance according to ESSQS certification status.

## 4 Discussion

In this randomized video‐review study, AI‐generated anatomical highlighting significantly improved surgeons' recognition of actual ureteral injuries in Videos A and C, both for overall recognition and for recognition within the predefined injury time‐code windows. These findings remained significant in post hoc sensitivity analyses based on the first recorded response and expanded time‐code windows. No significant between‐group difference was observed for pancreatic injury recognition in Video D, and correct judgments of no adverse events did not differ in Videos B and E. Overall, the results provide proof‐of‐concept evidence that AI‐generated overlays may support recognition of clinically important intraoperative adverse events under controlled video‐review conditions.

The present design cannot determine whether the observed improvement resulted from enhanced anatomical understanding, increased visual attention to the highlighted structure, or a combination of both. However, visual attention guidance may itself represent a clinically relevant function of AI‐assisted navigation. During surgery, attention is often concentrated on the immediate dissection site or instrument–tissue interaction. Highlighting a critical structure outside this primary focus may redistribute attention and support broader situational awareness of the operative field. This focus on human reader performance is consistent with prior studies showing that AI‐generated overlays can improve surgeons' organ recognition [15, 20]. Because eye tracking, attention allocation, and situational awareness were not directly measured, the proposed mechanism remains hypothetical.

To our knowledge, quantitative pixel‐level validation data for the specific ureter‐recognition model used in the present study have not been published. A related ureter‐recognition model developed within the EUREKA platform for intraoperative use during total hysterectomy reported a Dice similarity coefficient of approximately 0.51; however, that model was developed for gynecologic surgery and was not identical to the ureter model used in the present study [21]. A related pancreas‐recognition model developed within the EUREKA platform reported a Dice coefficient of 0.61 [13]. The pancreas model used in the present study belonged to the same model lineage but had subsequently been updated and was not identical to the previously reported model. Dice coefficients are generally lower for small or tubular anatomical targets, such as nerves and the ureter, than for larger, well‐demarcated organs, and should therefore be interpreted in the context of target morphology. These values provide general technical context and do not represent the segmentation performance of the specific models in the present video cohort.

For rare but serious surgical adverse events, conventional randomized trials using postoperative complication rates as endpoints may require prohibitively large sample sizes [7]. The counterfactual video‐review design used here compares AI‐enhanced and unenhanced versions of the same historical clinical event under controlled conditions, providing a feasible early‐stage reader‐performance framework. This design should be regarded as a proof‐of‐concept approach rather than evidence of general effectiveness across patients, surgeons, operative conditions, and injury patterns.

The primary and secondary recognition outcomes capture different aspects of performance. The primary outcome represents overall identification of the injured organ during or after video review, whereas recognition within the predefined injury time‐code window more specifically reflects temporal localization during the injury‐producing sequence. As illustrated in Figure 4, post‐window information included urine leakage from the thermally injured ureter in Video A, an anatomical inconsistency between the highlighted ureteral course and the dissection plane in Video C, and a cauterized area within the pancreatic parenchyma in Video D. Recognition recorded after the predefined window may therefore have reflected delayed recognition of secondary findings or retrospective inference from subsequent anatomical context. In contrast, recognition within the predefined injury time‐code window more directly reflects recognition during the injury‐producing sequence and may be more clinically relevant to opportunities for preventing or mitigating adverse events. However, the present study did not evaluate whether such recognition changed operative behavior or prevented injury.

Although the present study cannot establish that AI assistance would have changed the original operative course, recognition of an injury remains a prerequisite for timely confirmation and management. Video A involved a ureteral thermal injury that was not recognized intraoperatively and required postoperative ureteral stenting. The findings suggest that AI‐generated anatomical highlighting may support recognition in some comparable scenarios, but prospective intraoperative studies are required to determine whether such recognition alters surgical decisions or management.

For Video D, the overall risk difference was 6.7 percentage points, with a 95% confidence interval from −7.4 to 20.8 percentage points. This wide interval leaves substantial uncertainty and does not exclude a clinically meaningful benefit or a small unfavorable effect; the non‐significant result should therefore not be interpreted as evidence of no effect. In exploratory subgroup analyses, recognition was higher among non‐ESSQS‐certified surgeons in the EUREKA group, whereas among ESSQS‐certified surgeons the recognition rate was numerically lower in the EUREKA group than in the Normal group (70.4% vs. 84.6%), although this difference was not statistically significant. The latter pattern raises the possibility that overlays may occasionally distract experienced surgeons or interfere with established visual search patterns; however, this interpretation remains speculative.

Because participants were instructed to record an adverse event when they believed that an injury had occurred, the absence of an increase in incorrect adverse‐event judgments with AI enhancement suggests that judgment specificity was preserved under controlled video‐review conditions. However, this finding should not be interpreted as evidence of clinical safety. In actual surgery, cautious suspicion followed by inspection or confirmation may represent an appropriate safety behavior rather than an undesirable false alarm.

The use of recognition‐based intermediate outcomes is consistent with evaluation frameworks in other high‐risk fields, in which response time, attention allocation, cognitive load, and situational awareness may be assessed before reductions in rare adverse events can be demonstrated [22, 23]. In surgical AI, overall injury recognition, temporal localization of the injury‐producing event, and judgment in no‐injury situations may similarly provide complementary early‐stage measures.

This study has several strengths. First, it used actual intraoperative organ injury scenes rather than simulated or artificially created videos. Second, the same operative scenes were reviewed with or without AI‐generated overlays, allowing direct comparison under otherwise identical video conditions. Third, both injury and no‐injury videos were included. Fourth, reviewers were randomized and stratified according to laparoscopic colorectal surgical experience.

Several limitations should be acknowledged. First, this was a controlled video‐review study using isolated 2–3‐min clips and did not reproduce the cumulative temporal and spatial context, competing operative tasks, or real‐time decision‐making of actual surgery. Participants were explicitly asked to judge whether an adverse event had occurred, which may have increased vigilance compared with routine practice, although they were informed that both injury and no‐injury videos were included and the same adverse‐event judgment task was applied to both groups. Second, because no manually generated overlay or nonanatomical visual‐cue control was included, the study cannot distinguish enhanced anatomical understanding from visual saliency or attention redistribution. The specific ureter and pancreas model versions used in this study were not quantitatively evaluated against pixel‐level ground truth in the present video cohort.

Third, only three injury videos were evaluated, and each operative scenario was represented by a single case; the large number of reviewers does not eliminate the possibility of case‐specific effects. The no‐injury videos were selected by expert consensus as clinically comparable controls, but no formal difficulty‐matching scale was used, and perfect matching for local adiposity, inflammation, adhesions, anatomical exposure, visual obstruction, and tissue traction could not be ensured. Post‐window recognition may also have reflected secondary findings or retrospective inference. Prior experience with EUREKA or similar AI‐assisted navigation systems was not collected. Finally, the sample size was determined pragmatically, subgroup and interaction analyses were exploratory, and no adjustment for multiplicity was performed. Findings with *p* values near 0.05, particularly the exploratory Video D subgroup result, should be considered hypothesis‐generating. The wide confidence interval for Video D leaves open the possibility of Type II error. Most importantly, improved recognition under video‐review conditions does not establish changes in operative behavior, reduced organ injury rates, or improved patient outcomes.

In conclusion, AI‐generated anatomical highlighting improved surgeons' recognition of actual ureteral injuries under controlled video‐review conditions without increasing incorrect adverse‐event judgments. These findings provide proof‐of‐concept evidence that AI‐generated overlays may support timely recognition of clinically important intraoperative adverse events. Prospective intraoperative studies are needed to determine whether this recognition benefit facilitates earlier management, changes operative decision‐making, reduces complications, or improves patient outcomes.

## Author Contributions

**Shunjin Ryu:** conceptualization, funding acquisition, investigation, writing – original draft, methodology, validation, visualization, writing – review and editing, project administration, data curation, supervision. **Teppei Kamada:** formal analysis, methodology, data curation, validation, project administration, investigation. **Kai Neki:** data curation, methodology, validation, investigation. **Asuka Kuwashima:** methodology, resources. **Yuta Imaizumi:** data curation, investigation. **Shunsuke Nakashima:** data curation, investigation. **Yasuhiro Takano:** data curation, investigation. **Yasunobu Kobayashi:** data curation, investigation. **Yasuhiro Takeda:** data curation, investigation. **Ken Eto:** data curation, supervision, investigation.

## Funding

This study was supported by a grant from the Japanese Foundation for Research and Promotion of Endoscopy.

## Ethics Statement

This study was approved by the institutional review boards of Kawaguchi Municipal Medical Center (2024–46) and Machida Municipal Hospital (CT9‐2) and was conducted in accordance with the Declaration of Helsinki.

## Consent

Consent for research and educational use was obtained for all surgical videos. All videos and reviewer responses were anonymized.

## Conflicts of Interest

Shunjin Ryu has previously received research funding from Anaut Inc.; however, no funding from Anaut Inc. was received for the present study. Asuka Kuwashima is an employee of Anaut Inc. Statistical analysis was performed by Teppei Kamada, who had no financial relationship with Anaut Inc. Asuka Kuwashima was not involved in outcome adjudication, statistical analysis, or interpretation of the results. The other authors declare that they have no conflicts of interest.

## Supporting information


**Table S1:** Post hoc sensitivity analyses of organ injury recognition.

**Figure S1:** Representative scenes from the no‐injury control videos with and without AI‐generated anatomical highlighting.
The left column shows Video B, which depicts rectal dissection around the ureter without ureteral injury. The right column shows Video E, which depicts splenic flexure mobilization around the pancreatic tail without pancreatic injury. The upper row shows the original images, and the lower row shows the corresponding AI‐enhanced images. In Video B, the AI overlay highlights the ureter, whereas in Video E, it highlights the pancreas.

## Data Availability

The data that support the findings of this study are available on request from the corresponding author. The data are not publicly available due to privacy or ethical restrictions.
