# Supplementary material for: AI‐Assisted Recognition of Actual Ureteral and Pancreatic Injuries in Laparoscopic Colorectal Surgery: A Randomized Video Review Study
Source: Ann Gastroenterol Surg. 2026 Aug 24:10.1002/ags3.70267. Online ahead of print. doi: 10.1002/ags3.70267 (PMC13503624; doi:10.1002/ags3.70267)
Supplement: Supplementary file 1 [file AGS3-9999-0-s002.docx]

**Supplementary Table 1. Post hoc sensitivity analyses of organ injury recognition**

| **Video and outcome** | **EUREKA group (n=75)** | **Normal group (n=75)** | **Risk difference, percentage points (95% CI)** | **P value** |
| --- | --- | --- | --- | --- |
| **Video A (Ureteral injury during rectal dissection)** |  |  |  |  |
| Main recognition | 30 (40.0%) | 17 (22.7%) | +17.3 (2.9 to 31.8) | 0.022 |
| Recognition within the predefined injury time-code window | 15 (20.0%) | 4 (5.3%) | +14.7 (3.7 to 25.6) | <0.01 |
| Recognition within the expanded injury time-code window (114–128 s) | 16 (21.3%) | 5 (6.7%) | +14.7 (3.5 to 25.9) | <0.01 |
| Correct recognition based on the first-recorded response | 29 (38.7%) | 16 (21.3%) | +17.3 (2.7 to 31.1) | 0.021 |
| **Video C (Ureteral injury during difficult rectal dissection)** |  |  |  |  |
| Main recognition | 38 (50.7%) | 21 (28.0%) | +22.7 (7.4 to 37.9) | <0.01 |
| Recognition within the predefined injury time-code window | 14 (18.7%) | 3 (4.0%) | +14.7 (3.3 to 26.0) | <0.01 |
| Recognition within the expanded injury time-code window (107–129 s) | 15 (20.0%) | 6 (8.0%) | +12.0 (0.8 to 23.3) | 0.034 |
| Correct recognition based on the first-recorded response | 35 (46.7%) | 17 (22.7%) | +24.0 (8.8 to 37.7) | <0.01 |
| **Video D (Pancreatic injury during splenic flexure mobilization)** |  |  |  |  |
| Main recognition | 58 (77.3%) | 53 (70.7%) | +6.7 (−7.4 to 20.8) | 0.352 |
| Recognition within the predefined injury time-code window | 24 (32.0%) | 24 (32.0%) | 0.0 (−15.0 to 15.0) | 1.0 |
| Recognition within the expanded injury time-code window (113–127 s) | 40 (53.3%) | 31 (41.3%) | +12.0 (−3.9 to 27.1) | 0.191 |
| Correct recognition based on the first-recorded response | 57 (76.0%) | 53 (70.7%) | +5.3 (−8.8 to 19.2) | 0.46 |

The expanded injury time-code windows extended both the beginning and end of each predefined window by 2 seconds. Values are presented as the number of reviewers with the outcome and percentage.
